# Diphyllobothriasis from Eating Sushi

**DOI:** 10.4269/ajtmh.21-0105

**Published:** 2021-04-12

**Authors:** Yuya Ando, Yosuke Ono, Sachiko Ono

**Affiliations:** 1 Department of General Medicine, National Defense Medical College, Saitama, Japan;; 2 Department of Family Medicine, Graduate School of Medical and Dental Sciences, Tokyo Medical and Dental University, Tokyo, Japan;; 3 Department of Eat-loss Medicine, Graduate School of Medicine, The University of Tokyo, Tokyo, Japan

**Figure 1.**
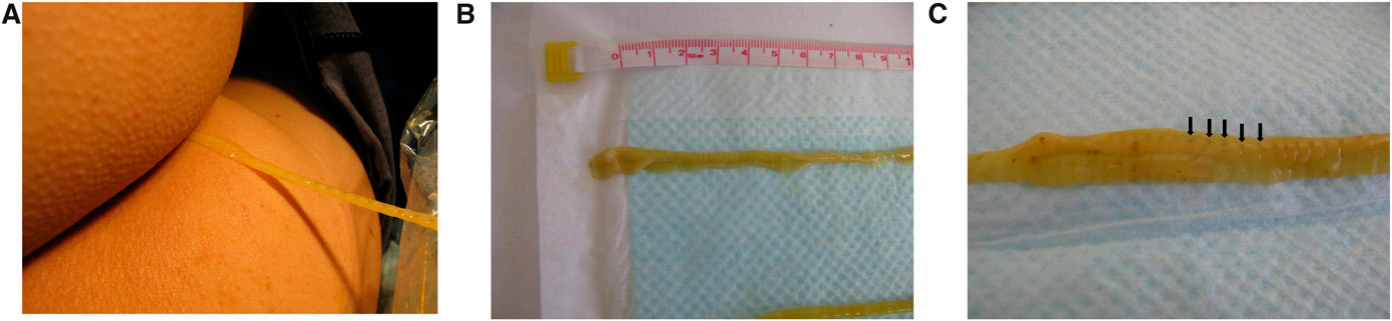
(**A**) A yellow, ribbon-like object being extracted from the patient. Caution was taken so that the object was not cut. The object was removed from the anus using a clear plastic bag while the patient was in the lateral position. (**B**) The yellow, segmented, ribbon-like object had a spoon-shaped head. The object was identified as a tapeworm. (**C**) The proglottid of the tapeworm. The black spots with arrows are considered the uterus; however, it was impossible to determine with the naked eye if the uterus contained eggs. This figure appears in color at www.ajtmh.org.

A healthy 20-year-old Japanese man presented to the hospital with a ribbon-like object protruding from his anus (Figure 1A). He frequently enjoyed eating sushi, and he especially loved raw salmon. He had never traveled outside of Japan. He reported bowel irritation, mild diarrhea, and 3 kg of weight loss over the course of the previous 3 days. Blood test results were unremarkable, and there was no evidence of anemia. He had been diagnosed with *Vibrio parahaemolyticus* infection the previous year after consuming uncooked whitebait.

On examination, he had a yellow, flat, ribbon-like object entangled in toilet paper protruding from his anus. The ribbon-like object, which was segmented and had a spoon-shaped head, was identified as a tapeworm (Figure 1B and 1C). The diagnosis was consistent with the patient’s preference for raw salmon; however, the identification of eggs or proglottids in the stool was required for a definitive diagnosis.

The patient was treated with a single dose of praziquantel while hospitalized; however, no eggs or segments of the worm were identified in his feces. It was surmised that the entire body of the worm and all eggs had been evacuated before hospitalization. His bowel irritation and mild diarrhea resolved, and he has not reported any recurrence since the time of discharge.

Diphyllobothriasis is a zoonosis caused by a particular genus of fish tapeworm acquired from ingesting infected raw or undercooked fish, especially in Japan; salmon infected with *Dibothriocephalus nihonkaiensis* is often implicated.^1^ Such cuisine is now prevalent worldwide, and it is currently estimated that more than 20 million people are currently infected with this cestodosis, making it the most common zoonosis worldwide.^2^ A recent taxonomic and genetic survey subdivided some members of the genus *Diphyllobothrium* into the genera *Dibothriocephalus*, *Adenocephalus*, and others.^3^

Because tapeworm infection sometimes causes vitamin B12 deficiency, information about previous ingestion of fresh or undercooked fish should be sought for patients with megaloblastic anemia.^3,4^ However, the diagnosis should be confirmed by checking for ova or proglottids in the patient’s stool.^2^ Additionally, broad tapeworms are sometimes found during colonoscopy.^5^

Tapeworm eggs have an operculum at one end, and the genital pore and uterus are situated in the center of each proglottid.^6^ Although we did not confirm the presence of eggs, based on the food preference of the patient, we believe this tapeworm was of the genus *Diphyllobothrium*.

Because globalization has resulted in the consumption of sushi and other forms of raw fish worldwide, and because the prevalence of zoonoses has increased, any clinician may encounter parasitic diseases.
